# Functional brain–heart interplay extends to the multifractal domain

**DOI:** 10.1098/rsta.2020.0260

**Published:** 2021-12-13

**Authors:** Vincenzo Catrambone, Riccardo Barbieri, Herwig Wendt, Patrice Abry, Gaetano Valenza

**Affiliations:** ^1^ Research Center E.Piaggio, Department of Information Engineering, School of Engineering, University of Pisa, Pisa, Italy; ^2^ Department of Electronics, Informatics and Bioengineering, Politecnico di Milano, Milano, Italy; ^3^ IRIT–ENSEEIHT, Université de Toulouse, CNRS, Toulouse, France; ^4^ University of Lyon, ENS de Lyon, University Claude Bernard, CNRS, Laboratoire de Physique, Lyon, France

**Keywords:** brain–heart interplay, multifractal spectra, maximal information coefficient, electroencephalography, heart rate variability, point process

## Abstract

The study of functional brain–heart interplay has provided meaningful insights in cardiology and neuroscience. Regarding biosignal processing, this interplay involves predominantly neural and heartbeat linear dynamics expressed via time and frequency domain-related features. However, the dynamics of central and autonomous nervous systems show nonlinear and multifractal behaviours, and the extent to which this behaviour influences brain–heart interactions is currently unknown. Here, we report a novel signal processing framework aimed at quantifying nonlinear functional brain–heart interplay in the non-Gaussian and multifractal domains that combines electroencephalography (EEG) and heart rate variability series. This framework relies on a maximal information coefficient analysis between nonlinear multiscale features derived from EEG spectra and from an inhomogeneous point-process model for heartbeat dynamics. Experimental results were gathered from 24 healthy volunteers during a resting state and a cold pressor test, revealing that synchronous changes between brain and heartbeat multifractal spectra occur at higher EEG frequency bands and through nonlinear/complex cardiovascular control. We conclude that significant bodily, sympathovagal changes such as those elicited by cold-pressure stimuli affect the functional brain–heart interplay beyond second-order statistics, thus extending it to multifractal dynamics. These results provide a platform to define novel nervous-system-targeted biomarkers.

This article is part of the theme issue ‘Advanced computation in cardiovascular physiology: new challenges and opportunities’.

## 1. Introduction

Several biochemical, anatomical and functional links form dynamic connections between the central nervous system (CNS) and the autonomic nervous system (ANS), with these anatomical and functional connections referred to as the central autonomic network (CAN) [1,2].

Information relating to CAN processes sent by higher-order cortex regions is influenced by the environmental context together with afferent signals from visceral receptors (i.e. pressure, chemical, mechanical and temperature), and manifests as various reflexes and autonomic responses [1,3]. Considering a variety of physical interactions between the cerebral and cardiovascular systems, numerous studies have attempted to characterize CAN activity and the associated functional brain–heart interplay (BHI). In particular, focusing on electroencephalography (EEG) and heart rate variability (HRV) series, several studies have investigated CAN-related activity through linear and nonlinear approaches, including information transfer [4,5], nonlinear convergent cross mapping [6], maximal information coefficient [7,8], joint symbolic analysis [9], Granger causality indices [10,11] and ad hoc functional models [12,13].

Although nonlinearity and non-stationarity in physiological systems dynamics can be due to interactions among system subcomponents [14], heartbeat and EEG dynamics also exhibit multifractal (MF) behaviour [15–19]. Specifically, heartbeat and EEG series have transient and local non-Gaussian structures as well as multiple local singular behaviours that exceed self-similarity [15,18,20,21], each of which can be characterized using a specific Hurst exponent H. To describe these singular behaviours comprehensively and identify transient self-similar processes, a collection of exponents H=h(t) and a *multifractal spectrum* D(h) are required. Different methodologies have been proposed to estimate the MF spectrum: MF detrended fluctuation analysis [22], the wavelet transform modulus maxima method [23], the wavelet leader MF formalism [24] and its generalization using p-leaders [25,26]. The wavelet p-leader MF formalism leads to a non-Gaussian expansion for characterizing the temporal dynamics. Recently, we exploited this formulation to describe various brain and heartbeat dynamics during a cold pressor test (CPT) [27–30].

Regarding cardiac autonomics, a CPT evokes a strong sympathovagal change following the immersion of a distal limb (e.g. hand or foot) in iced water (approx. $4^{\circ }\text{C}$). In particular, CPTs provoke an increase in sympathetic nerve activity and plasma norepinephrine within the muscles, which is thought to be driven by nociceptive fibres, leading to heightened blood pressure and an increase in the concentration of venous norepinephrine [31,32]. Studies on healthy subjects have reported that CPTs increase both blood pressure and vessel peripheral resistance [31]. Furthermore, it has been argued that heart rate stationarity during CPTs results from the sequence of a decreasing phase following an initial increase [32]. Although the use of CPTs, both in clinics and research, was previously limited purely to sympathetic elicitation [32], more recent analyses of HRV fractal features demonstrated cardiac autonomic co-activation within the vagal and sympathetic systems [27,33]. Regarding EEG dynamics, CPTs provoke changes in the frontal lobes that are caused by oscillations in the δ and θ bands [28], as well as posterior-parietal activity in the α band, and peripheral bilateral temporal regions in the β range [34]. With respect to a resting state in healthy subjects, CPTs are associated with a reduction in MF features both for EEG [28] and heartbeat dynamics [27].

While MF behaviour has been characterized in brain and cardiovascular systems separately, the eventual and possibly synchronized co-occurrence of MF changes in brain and heartbeat dynamics has yet to be investigated, and could highlight the complexity of the BHI phenomenon. Therefore, in this study, we describe a novel methodological framework to extend the study of functional BHI to the MF domain. The proposed signal processing framework combines MF features extracted at different time-scales from brain and heartbeat dynamics, while the maximal information coefficient (MIC) is used to quantify the related functional BHI. The performance of the framework is evaluated using real EEG and heartbeat data gathered from 24 healthy participants during a CPT session. Preliminary results of the proposed study have been recently reported in [35].

## 2. Material and methods

### (a) Nonlinear multiscale (mutifractal-inspired) representations

Multiscale representations are classically achieved by computing wavelet coefficients, $d_{X}(j,k)=\langle \psi_{j,k}|X\rangle$, obtained by comparing through inner product the signal or time series to analyse, X(t), against a collection of dilated and translated templates $\psi_{j,k}$ of a reference pattern called the mother wavelet $\psi_{0}(t)$ (cf. e.g. [36]). These multiscale representations have been classically used to characterize scale-free temporal dynamics in biomedical applications (cf. e.g. [18–20,37,38]). Notably, self-similarity, a reference model for scale-free dynamics, can be assessed and quantified via a power-law behaviour with respect to scales of the so-called wavelet spectrum (cf. e.g. [39]):

2.1 $$S_{d_{X}}(j,2)=\frac{1}{n_{j}}\sum_{k=1}^{n_{j}}|d_{X}(j,k){|}^{2}≃K2^{j2H},$$

with $n_{j}$ the number of wavelet coefficients available at scale $2^{j}$, H the self-similarity exponents, and ≃ indicating that the sample moment has a power law behaviour with exponent 2H for a large range of scales $2^{j}$. Analogous to the Fourier transform, $S_{d_{X}}(j,2)$ estimates the energy distribution in the frequency domain and, consequently, the Hurst parameter H refers only to linear properties of the data [37,39].

Multifractality has also been used to model scale-free dynamics beyond energy repartitions, and hence beyond the second-order statistics. To assess multifractality in temporal dynamics, it has been shown that a new multiscale representation must be built by replacing wavelet coefficients with novel multiscale quantities referred to as the wavelet p-leaders $\ell_{X}^{\left(p\right)}$. These quantities are defined as local $\ell^{p}$ norms of wavelet coefficients in a narrow temporal neighbourhood over all finer scales:

2.2 $$\ell_{X}^{\left(p\right)}(j,k)=(2^{j}\sum_{\lambda^{\prime }\subset 3\lambda_{j,k}}2^{-j^{\prime }}|d_{X}(\lambda^{\prime }){|}^{p}{)}^{1/p},$$

with $\lambda_{j,k}=[k2^{j},(k+1)2^{j})$ and $3\lambda_{j,k}=\underset{m\{-1,0,1\}}{⋃}\lambda_{j,k+m}$ [24–26]. Multifractality can be quantified by considering that wavelet p-leaders moments of positive and negative orders q (in contrast with the second-order moments only as in equation (2.1)) behave as power laws with respect to:

2.3 $$S_{\ell_{X}^{\left(p\right)}}(j,q)=\frac{1}{n_{j}}\sum_{k=1}^{n_{j}}|\ell_{X}^{\left(p\right)}(j,k){|}^{q}≃K_{q}2^{j\zeta (q)}.$$

The collection of scaling exponents ζ(q) for positive and negative q can be associated with the *multifractal spectrum* D(h) of the signal X, and thus describes fine details of its temporal dynamics.

A first limitation in the use of these scaling exponents ζ(q) lies in the fact that they mix-up linear and nonlinear temporal dynamics. To disentangle nonlinear dynamics from linear dynamics, it has been proposed to rewrite $S_{\ell_{X}^{\left(p\right)}}(j,q)=(1/n_{j})\sum_{k=1}^{n_{j}}|\ell_{X}^{\left(p\right)}(j,k){|}^{q}$ as $S_{\ell_{X}^{\left(p\right)}}(j,q)=(1/n_{j})\sum_{k=1}^{n_{j}}\exp(q\log|\ell_{X}^{\left(p\right)}(j,k)|)$ thus motivating the use of the cumulants of log-leaders [24], defined as

2.4 $$C_{m}^{\left(p\right)}(j)\equiv {\text{Cum}}_{m}\log(\ell_{X}^{\left(p\right)}(j)).$$

Indeed, for scale-free dynamics, these cumulants behave as $C_{m}^{\left(p\right)}(j)=c_{m}^{0}+c_{m}\log(2^{j})$, and it can further be shown that $C_{1}(j)$ and $c_{1}$ are associated with the location of the maximum of multifractal spectrum, while $C_{2}(j)$, $C_{3}(j)$ and $C_{4}(j)$ can be associated with its width, asymmetry and flatness [24]. This indicates that $C_{1}(j)$ quantifies the linear (or second-order-statistics) temporal dynamics of the signal, while $C_{m}(j)$ for m≥2 are related to higher order statistics and nonlinear dynamics. The cumulants $C_{m}(j)$ for m≥2 quantify departures from Gaussianity as a function of scales $2^{j}$ for the distributions of the $\log|\ell_{X}^{\left(p\right)}(j,k)|)$ which are thus used as quantifiers of the nonlinear temporal dynamics of the signal X.

A second limitation further stems from assuming exact scale-free dynamics and power-law behaviours of these statistics as a function of the scales. Thus, instead of assuming *a priori* such power laws and extracting scaling exponents ζ(q) or $c_{m}$, one can use such multiscale representations as a function of scales $2^{j}$.

To account for both limitations, focusing on nonlinear dynamics only and by not assuming *a priori* exact scale-free dynamics, it has been proposed to construct new multiscale quantities that focus on some aspects of the non-Gaussianity of the data, $L_{q}^{2P}(j)$, from log-cumulants beyond order 1 [40]:

2.5 $$L_{q}^{2P}(j)=\sum_{m=2}^{\infty }C_{m}(j)\frac{\sum_{i=1}^{P}q_{2i-1}^{m-1}-q_{2i}^{m-1}}{m!}.$$

Because $C_{1}(j)$ does not enter in (2.5), $L_{q}^{2P}(j)$ defines a purely nonlinear data feature. In addition, the choice of the moments $q_{i}$ allows us to tune the sensitivity of the multiscale representation to different departures from Gaussianity (see [40] for further details), because they effectively act as weights for the cumulants at orders as in equation (2.5). Table 1 shows a list of the three choices, defining the $L_{q}^{2P}(j)$ used in this study: $\text{LQ1}\equiv L_{q}^{\left(2\right)}(j)$ is sensitive to any form of departures from non Gaussianity and involves positive moments only, thus all cumulants are contributing to (2.5), hence *active*; $\text{LQ2}\equiv L_{q}^{(2)*}(j)$ also involved two different moments, yet positive and negative, so that the resulting multiscale representation is sensitive to departures from Gaussianity that are symmetric only (only even cumulants contribute to (2.5)); $\text{LQ3}\equiv L_{q}^{\left(4\right)}(j)$ combines four moments in such a way that the second cumulant $C_{2}(j)$ does not contribute, so that LQ3 focuses on departures from Gaussianity not well modelled by long-normality (only cumulants for m≥3 contribute to (2.5)). Further details are reported in [40].

**Table 1.** Non-Gaussian expansion indices.

notation	moments $q_{i}$	cumulants $C_{m}$ active in (2.5)
$\mathrm{LQ}1\equiv L_{q}^{\left(2\right)}(j)$	(0.25,2)	m≥2
		any departure from Gaussian
$\mathrm{LQ}2\equiv L_{q}^{(2)*}(j)$	(−2,2)	m=2,4,…
		symmetric properties
$\mathrm{LQ}3\equiv L_{q}^{\left(4\right)}(j)$	(0.25,0.75,2.5,2))	m≥3
		non-lognormal non-Gaussian

### (b) Derivation of electroencephalography power spectra

EEG series were preprocessed following the so-called HAPPE procedure, described in [41]. Briefly, from the 128 original electrodes, the most peripheral 38 channels were discarded, leaving 90 electrodes, to avoid overlearning in the following independent component analysis (ICA) [41]. A bad-channel rejection was applied to derive the normed joint probability of the average logarithmic power in the range of 1–125 Hz, with the actual rejection applied to those channels external to the 1% tails of the distribution. The removed channels were subsequently spherically interpolated, thereby exploiting neighbouring EEG series. Next, EEG signals were then band-pass filtered between 0.5 Hz and 100 Hz; the electrical noise at 50 Hz and its first harmonic was rejected by applying a multitaper regression method [41]. Artefact recognition and correction was then performed for the EEG series using a wavelet-enhanced ICA algorithm, which focuses on eye- or muscle-related artefacts in the recording. Further artefacts were recognized using a machine-learning-based algorithm for analysing independent components [41]. Following this, the EEG series were re-referenced to the time-varying common average among all electrodes. Eventually, all time series were visually inspected by an expert operator.

For each EEG electrode, the power spectral density (PSD) was then estimated by implementing the Welch method (with 2 s Hamming window and 0.25 s moving step); thus, the resulting time-frequency representation was sampled at 4 Hz. The PSD time course was then filtered within the five standard frequency bands: δ∈[1−4], θ∈[4−8], α∈[8−12], β∈[12−30] and γ∈[30−70] (all expressed in Hz). Consequently, for each of the 90 selected EEG channels (ch), we ended up with five time series (one for each frequency band): $X_{\nu ,\text{ch}}(t_{h})={\text{PSD}}_{\nu ,\text{ch}}(t_{h})$, where $t_{h}$ is the time instant on which the sliding Hamming window is centred and ν∈δ,θ,α,β,γ.

### (c) Point-process model for heartbeat dynamics

Heartbeat dynamics series were derived from standard electrocardiogram (ECG) processing. The Pan–Tompkins algorithm was implemented to locate the R-peak events temporally, and algorithmic and physiological artefacts were identified and corrected by employing point-process-based statistics [42].

In previous studies, we demonstrated the crucial role of heartbeat preprocessing in MF estimation and found that inhomogeneous point-process models perform better than commonly used non informative interpolations [27,38]. The point-process (PP) framework defines the probability of a heartbeat event in the continuous-time domain. Such a probability function accounts for the instantaneous estimation of features that are appropriate for short-time physiological variations. Formally, defining t∈[0,T], the observation period, and $0\leq u_{1}<\cdots <u_{k}<u_{k+1}<\cdots <u_{K}\leq T$ as the R-wave event times, one can define $N(t)=\max\{k:u_{k}\leq t\}$ as the sample path of the related counting process. Extracting its derivative (dN(t)) defines a continuous-time indicator function, which equals 1 (dN(t)=1) when a ventricular contraction event is detected, and is null (dN(t)=0) otherwise. Defining $H_{t}=(u_{j},{\text{RR}}_{j},{\text{RR}}_{j-1},\ldots ,{\text{RR}}_{j-M+1})$ as the sequence of events, the PP framework defines an inverse Gaussian probability density function (PDF) for the time $t-u_{j}$ until the next ventricular contraction [43]. This is described by the following mathematical formulation:

2.6 $$f(t|H_{t},\xi (t))={\left[\frac{\xi_{0}(t)}{2\pi {\left(t-u_{j}\right)}^{3}}\right]}^{1/2}\times \exp\left\{-\frac{1}{2}\frac{\xi_{0}(t){\left[t-u_{j}-\mu_{\text{RR}}(t,H_{t},\xi (t))\right]}^{2}}{\mu_{\text{RR}}{\left(t,H_{t},\xi (t)\right)}^{2}(t-u_{j})}\right\},$$

where $j=\tilde{N}(t)$ is the index of the previous R-wave preceding time t, ξ(t) is the vector of the parameter time course, $\mu_{\text{RR}}(t,H_{t},\xi (t))$ is the first-order moment (mean) of the PDF, and $\xi_{0}(t)>0$ is the inverse Gaussian PDF shape parameter [43]. The function $f(t|H_{t},\xi (t))$ represents the probability of a heartbeat occurring at time t, based on the knowledge that the previous heartbeat occurred at time $u_{j}$; thus, $\mu_{\text{RR}}(t,H_{t},\xi (t))$ can be considered as the expected time until the following heartbeat occurs. The formulation $f(t|H_{t},\xi (t))$ as a time-varying inverse Gaussian PDF is motivated by a resemblance to the physiological behaviour and by goodness-of-fit comparisons [43].

In this study, we used the same signal processing framework described in [44,45]. Briefly, heartbeat dynamics were processed to derive instantaneous series defined in the time, frequency and nonlinear/complex domains; we derived instantaneous linear estimates in the time domain as first- (mean) and second-order (variance) moments of the PDF (e.g. $\sigma_{\text{RR}}^{2}$) [43], as well as instantaneous frequency-domain estimates based on the linear power spectrum. Following the mathematical details reported in [44], it is then possible to obtain the instantaneous spectral power in the low-frequency (LF=0.05–0.15 Hz) (*powLF*) and high-frequency (HF=0.15–0.5 Hz) (*powHF*) bands. Furthermore, the instantaneous LF and HF power ratio is used as a feature linked to the sympathovagal balance (LF/HF). Third, aiming to characterize heartbeat dynamics beyond the second-order moment, higher-order spectra (HOS) were derived to account for phase relations among the spectral components [46]. Formally, HOS are defined as statistics moments and higher-than-third-order cumulants, which can be used to study and detect phenomena that overcome classical assumptions of linearity, stationarity and Gaussianity [44,46]. HOS generally represent an analysis framework that is virtually extensible to any Nth-order statistics. In this study, we extracted the most commonly used HOS estimate, which is the third-order spectrum (also referred to as the bispectrum), by exploiting the mathematical formulation proposed in [44] as it allows for a dynamical estimation. Following this approach, we integrated the bispectrum in three different areas: on both dimensions in the LF range (obtaining *LL*), on both dimensions in the HF range (obtaining *HH*), and in the area corresponding to the LF range on the x-axis and the HF range on the y-axis (obtaining *LH*). Fourth, to account for the complex dynamics of the cardiovascular system, we extracted two additional features: the Lyapunov exponent (*Lyap*) in a time–varying shape, which follows the formulation proposed in [47] and exploits a cubic autoregressive formulation, and a sample entropy estimation *pSamEn* embedded in the inhomogeneous PP nonlinear framework [44]. We determined the optimal model orders using the Kolmogorov–Smirnov (KS) statistics in the *post-hoc* analysis [43], and set M=9.

Finally, all PP-derived features were resampled at 4 Hz.

### (d) Maximal information coefficient

The MIC quantifies the linear and nonlinear dependencies that exist between a pair of samples. It descends directly from the scatterplot of the two series being coupled (x and y) [48]. Indeed, a grid, comprising any number of rows and columns, can be superimposed on the scatterplot. Considering all the possible partitions of such a grid, we form the ensemble $G_{n_{x}\times n_{y}}$, where $n_{x}$ and $n_{y}$ are the numbers of rows and columns, respectively; the algorithm estimates the mutual information ($I_{g}$) associated with all elements $g\in G_{n_{x}\times n_{y}}$ and extracts the maximum among $G_{n_{x}\times n_{y}}$:

2.7 $$m_{n_{x}\times n_{y}}=\frac{\underset{g\in G_{n_{x}\times n_{y}}}{\max\left\{I_{g}\right\}}}{\log\min\{n_{x},n_{y}\}}.$$

Then, the MIC is derived as the maximal $m_{n_{x}\times n_{y}}$ over all possible pairs $\left(n_{x},n_{y}\right)$. Another possible estimation of the MIC was found to be $\text{MIC}(x,y)=\max_{n_{x}n_{y}<B}\{m_{n_{x}\times n_{y}}\}$, with B empirically defined as $B=n^{0.6}$ (see [48] for a more in-depth explanation).

We selected the MIC, as opposed to other measures of dependence (e.g. Pearson linear correlation coefficients), as it allows for the quantification of a wider genre of dependencies (linear and nonlinear) [48], and has been used before in BHI estimation studies [7,8].

### (e) Coupled brain–heart multifractal quantities

In previous studies, we made the following observations:

— Both the brain and the cardiovascular systems show intrinsic MF dynamics [27,28],
— MF features derived from heartbeat dynamics are more reliable when a point-process model is applied [27],
— The MIC is a reliable tool for assessing linear and nonlinear coupling between CNS and ANS dynamic series [7,8].

Building upon these experimental findings, we devised a novel processing pipeline that combines this knowledge with the aim of studying BHI in the MF domain and avoiding making a strong hypothesis on the BHI dynamics.

**Figure 1.**
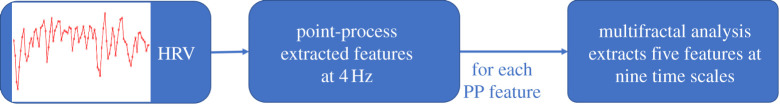
Schematic of the HRV signal processing procedure. Point-process extracts linear, nonlinear, bispectral and complexity features. (Online version in colour.)

**Figure 2.**
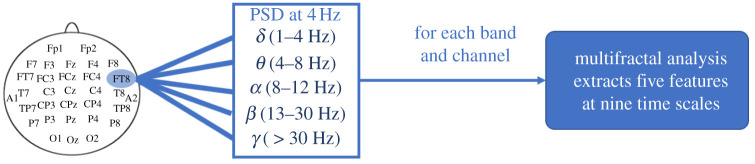
Schematic of the EEG signal processing procedure. (Online version in colour.)

The procedure followed for the heartbeat analysis is summarized in figure 1. Similarly, figure 2 illustrates the procedure applied in the EEG series analysis. Both the EEG–PSD series and the HRV-derived feature time courses require resampling at 4 Hz in order to provide a coherent comparative analysis.

**Figure 3.**
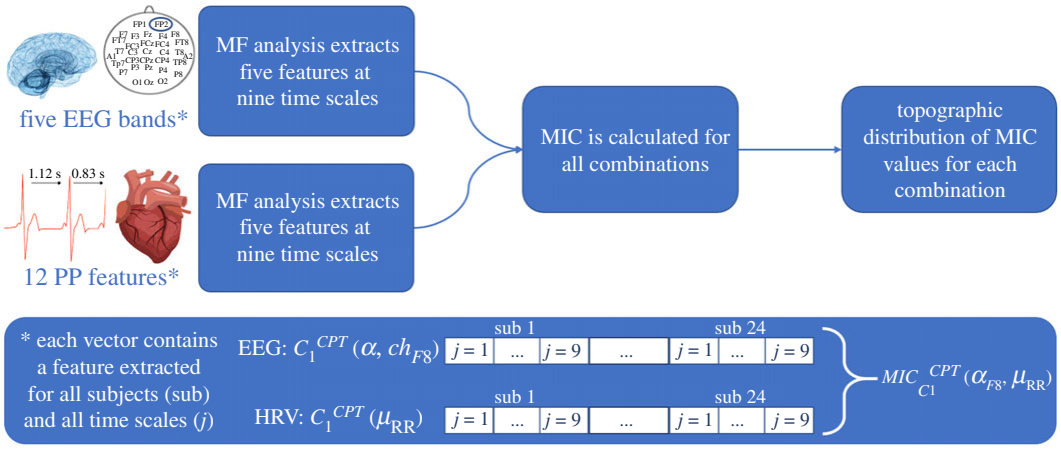
Schematic of the proposed signal processing framework. (Online version in colour.)

The processing pipeline continues with the implementation of the MF analysis. We extracted the first- and second-order cumulants from the MF spectrum (i.e. C1(j,s) and C2(j,s)) and three non-Gaussian expansion terms (namely, LQ1(j,s),LQ2(j,s), and LQ3(j,s)), at all time scales permitted by the length of the signals (i.e. j=1,…,9 corresponding to time scales of [0.6667,1.3333,2.6667,5.3333,10.6667,21.3333,42.6667,85.3333,170.6667] seconds, respectively), from all subjects (i.e. s=1,…,24). Consequently, the analysis provides five indexes, which vary with respect to the time scales and subjects, and for each time series: EEG–PSD from all channels, and HRV-derived measures. To measure the linear and nonlinear coupling between the brain and heart MF measures, the MIC implementation was used. This step is supposed to be applied in a multi-subject experimental condition, thus allowing the multiscale MF measures contributed by different subjects to concatenate (figure 3); this operation increases the statistical robustness of the MIC [48].

The steps outlined above result in a collection of MIC values that build a topographical distribution of MIC estimations across the scalp. At this stage, the variability across EEG bands, different HRV measures and MF quantities are maintained. All the topographical distributions, for all combinations of features and for both CPT and resting state, are reported in the electronic supplementary material.

The functional relationship between brain and heart MF dynamics was estimated by applying the MIC at a group level. Thus, the functional BHI was quantified as ${\text{MIC}}^{\text{ph}}(x_{\text{MF}},y_{\text{MF}})$, where the superscript ph represents the experimental phase (i.e. rest or CPT), MF represents the MF feature being analysed (i.e. one of C1,C2,LQ1,LQ2 or LQ3), and the vectors $x_{\text{MF}}$ and $y_{\text{MF}}$ represent the collection of MF estimates derived at all nine time scales for each subject. More specifically, $x_{\text{MF}}$ represents the array of estimates associated with a feature from the ANS side (e.g. $\text{C1}(\mu_{\text{RR}})$) and $y_{\text{MF}}$ denotes an array of MF estimates associated with a feature from the CNS dynamic at a specific frequency band and from an EEG channel (e.g. $\text{C1}({\text{PSD}}_{\alpha ,Fp_{2}})$, where $Fp_{2}$ represents the channel). In other words, the two vectors used to derive the MIC ($x_{\text{MF}}$ and $y_{\text{MF}}$) consist of the collection of the same MF estimates extracted from a given heart- and brain-derived feature for all time scales and subjects. For example, the coupling extracted with ${\text{MIC}}_{\text{C1}}^{\text{CPT}}(\mu_{\text{RR}},\theta_{F8})$ refers to the dependencies between the first-order MF cumulant C1 calculated for $\mu_{\text{RR}}$ (for all time scales and subjects) and the θ-PSD of channel F8 (for all time scales and subjects) during the CPT.

**Figure 4.**
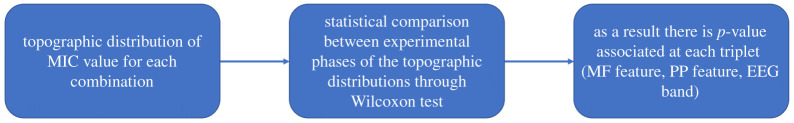
Schematic of a statistical comparison in the proposed processing pipeline. (Online version in colour.)

Finally, statistically significant differences between the topographic distributions of MIC values (across the 90 EEG channels) extracted for the two experimental conditions (i.e. rest or CPT) were investigated using Wilcoxon non-parametric tests for paired samples (a schematic of the implemented statistics is provided in figure 4) for each MF feature separately and for each combination of EEG frequency band and PP-derived feature. The use of non-parametric tests is justified by the non-Gaussian sample distribution as the MIC can take positive values in the [0,1] range only [48].

To summarize, considering the CNS, we used 90 EEG channels and five estimated PSD time series, while for the ANS, we derived the time course of the 12 PP features listed in table 2. For each of the 90 EEG channels, five EEG frequency bands, 12 PP-derived vectors and five MF features, a value of MIC was extracted between a pair of vectors, each containing 216 elements. These elements corresponded to the number of subjects (i.e. 24) times the number of time scales employed (i.e. nine). Statistical comparisons were performed between the two 90-elements vectors (i.e. the number of EEG channels) extracted during the CPT elicitation and rest states, respectively. Thus, a different p-value was acquired for each combination (triplet) of MF measure, EEG band and HRV feature (figure 4).

**Table 2.** List of features extracted from the inhomogeneous point-process model of heartbeat dynamics. BS, bispectrum.

	feature	explanation
nonlinear	HH	two-dimensional integral from BS estimation in bands (HF, HF)
dynamics	LH	two-dimensional integral from BS estimation in bands (LF, HF)
	LL	two-dimensional integral from BS estimation in bands (LF, LF)
	Lyap	largest Lyapunov exponent
	pSamEn	continuous estimation of sample entropy
linear	powLF	PSD extracted in the LF band [0.04−0.15) Hz
dynamics	powHF	PSD extracted in the HF band [0.15.0.4) Hz
	LF/HF	ratio between power in the LF and HF band
	$\mu_{\mathrm{RR}}$	first-order moment of the estimated continuous R-R series
	$\sigma_{\mathrm{RR}}^{2}$	second-order moment of the estimated continuous R-R series
	$\mu_{\mathrm{HR}}$	first-order moment of the estimated continuous HR series
	$\sigma_{\mathrm{HR}}^{2}$	second-order moment of the estimated continuous HR series

To account for multiple comparisons, we corrected the significance threshold according to the Bonferroni rule: we considered a total of 60 comparisons (five EEG\ bands×12 PP\ features), which yielded $\alpha =\alpha^{1}/60=0.00083$ (α being the significance threshold, with $\alpha^{1}=0.05$ chosen as the initial value).

### (f) Experimental set-up

High-density 128-channel EEG and single-lead ECG were collected from 30 healthy subjects (26.7 yr on average, gender balanced) at a sampling rate of 500 Hz. Signal acquisition was performed using a Geodesic NA300 EEG System (Electrical Geodesics Inc.). All participants volunteered to take part in the study and provided their informed consent. They asserted to be healthy and right-handed.

**Figure 5.**
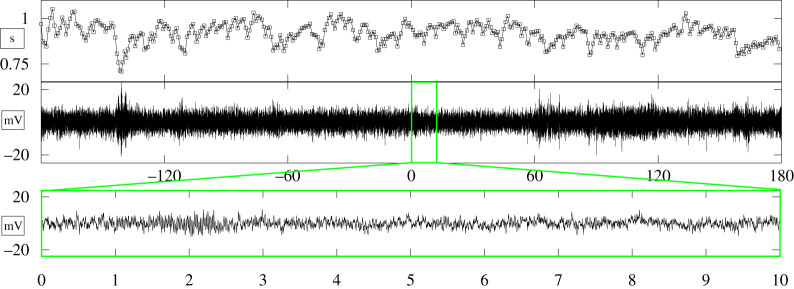
Exemplary HRV (top panel) and EEG (central panel) series from one representative subject. The bottom panel shows the first 6 s of the CPT. The EEG series was recorded from channel 33 over the left-central lobe. (Online version in colour.)

The experimental protocol consisted of an initial rest phase lasting 3 min and a CPT phase, in which subjects were instructed to submerge their left hand in iced water. Participants were requested to keep their hand submerged for up to 3 min but were free to stop the session if they experienced undue discomfort. A time threshold of 3 min was chosen in accordance with the literature on pain arousal in response to temperature stress [32]. Figure 5 shows exemplary EEG and HRV series gathered from one representative subject.

The local ethics committee, Area Vasta Nord-Ovest Toscana, approved all experimental procedures. Recordings from six participants were discarded either because of an early withdrawal from the CPT session, or because of significant movement-related artefacts. For further information, refer to [12].

## 3. Experimental results

**Figure 6.**
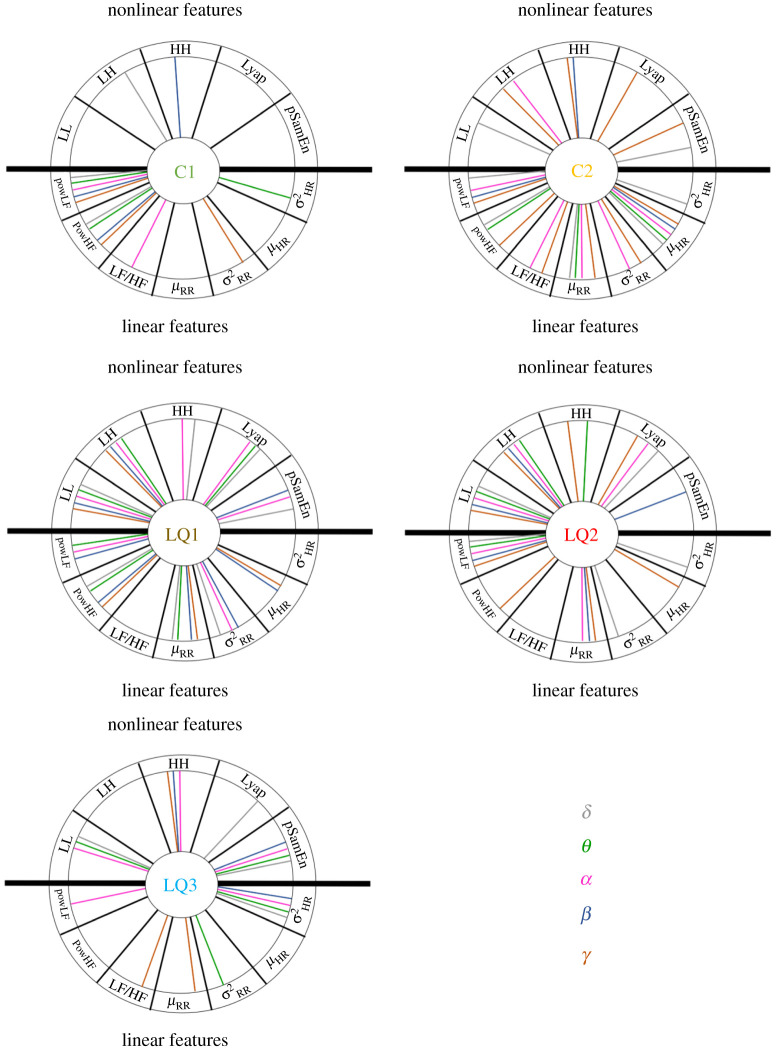
Schematic of statistically significant differences between the experimental conditions regarding the topographic distribution of MIC values. Each subfigure represents a single MF feature, whereas different colours refer to different EEG bands. (Online version in colour.)

The results are represented in figure 6, in which each subfigure refers to a single MF feature (i.e. C1, C2, LQ1, LQ2 and LQ3). The external circle was divided into 12 sections, one for each PP feature; the upper semi-plan contains features associated with nonlinear dynamics (i.e. *LL, LH, HH, Lyap, pSamEn*), and the lower semi-plan contains features associated with linear dynamics (i.e. *powLF, powHF, LF/HF*, $\mu_{\text{RR}},\sigma_{\text{RR}}^{2},\mu_{\text{HR}}$ and $\sigma_{\text{HR}}^{2}$). The five EEG frequency ranges (i.e. δ,θ,α,β and γ) are represented by different colours. In specific sections, a coloured segment linking the centre of a subfigure to the circumference indicates that the topographic distribution across the scalp corresponding to an MIC calculated for that specific MF feature (identified by the subfigure), PP-feature (identified by the section in the circle) and EEG band (identified by the colour) combination revealed statistically significant differences (i.e. corrected p-value<0.05) between the two experimental conditions.

The two upper panels in figure 6 show the results of the statistical comparison between the topographic distributions of the first- and second-order cumulants calculated over the MF spectrum. Many EEG band and PP feature combinations are significant, meaning that the functional relationship between brain and heart dynamics changes from the resting to the CPT phase from the MF perspective. The HRV–PSD quantities extracted in the LF and HF bands seem to be the most significant indices involving the first cumulant C1 (top-left panel); this is particularly evident from the power in the LF band. Both the C1 and C2 panels appear to display considerably closer connections with their corresponding lower semi-plan (referring to linear HRV-feature dynamics) compared to the upper semi-plan. Therefore, we can argue that the functional brain–heart coupling in the MF domain is driven mainly by the MF properties of instantaneous heartbeat estimates of linear dynamics.

The C2 panel in figure 6 (top-right) shows more significant connections than C1. In particular, almost all the HRV measures are coupled with the EEG γ band, followed by the α range, which shows significance when coupled with high-frequency estimations from the bispectrum of the HRV. Additionally, important connections are observed with the γ and β bands, and the EEG δ band, which exhibits significant couplings, particularly for the HRV features related to linear dynamics. By contrast, the θ band of the EEG is least represented.

Inspection of the LQ1 index, which takes into account the general non-Gaussianity of a time series, highlights many significant combinations and is represented in the central-left corner of figure 6. All the EEG bands seem to be involved, with few heartbeat indexes conspicuous owing to their insignificance (i.e. the LF/HF ratio and the variance of the HR). In this case, the EEG frequency range exhibiting the greatest coupling to HRV features is the β band, thereby confirming the presence of important couplings at these brain activity frequencies. The EEG θ band shows slight significance when coupled with features extracted from the bispectrum and the Lyapunov exponent; however, it is not coupled with most of the features extracted from the linear dynamics domain. It should be noted that the coupling of the Lyapunov exponent time series of the HRV is significantly different between the CPT and resting states at lower EEG frequencies, up to the α band, and this coupling is not effective when coupled with higher frequency ranges. The bispectral coefficients (i.e. HH, LH and particularly LL) that provide significant comparisons with both LQ1 and LQ2 (both central panels) are especially important. The LQ2 index considers the symmetry of the non-Gaussian distribution of the MF spectrum and is evidently different in its topographic distribution between the two experimental conditions, particularly for the HRV PSD in the LF band, and the bispectral quantities coupled with many EEG bands. The θ frequency range of the brain activity is less involved in this analysis. The third non-Gaussian index LQ3, represented in the bottom-left panel, shows significant couplings, albeit fewer than the previous two.

**Figure 7.**
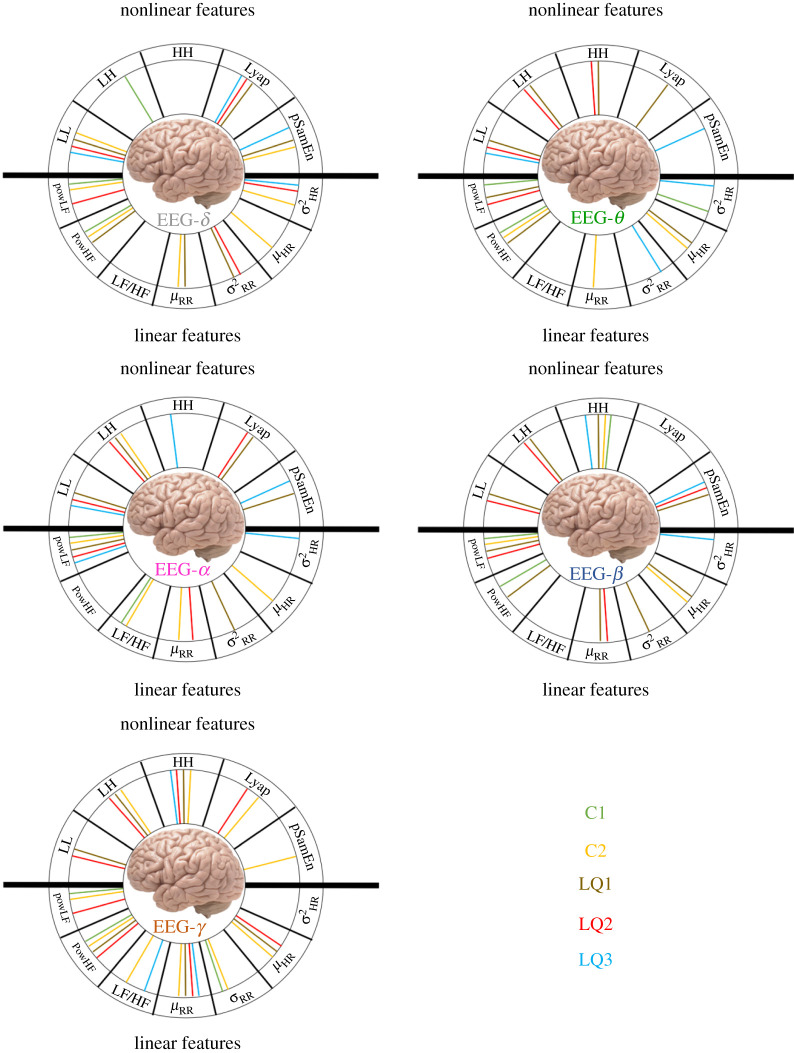
Schematic of statistically significant differences between the experimental conditions over the topographic distributions of MIC values. Each subfigure represents a single EEG band, with different colours referring to different MF features. (Online version in colour.)

The aforementioned results are also shown in figure 7, in which significant brain–heart MF connections are categorized according to EEG bands, while the different colours characterizing the linking segments represent the MF features. This figure shows that oscillations in the γ band (bottom-left panel) are connected most densely, including different MF and PP features (apart from $\sigma_{\text{HR}}^{2}$), with 30 significant couplings identified. Conversely, the θ band shows the lowest number of significant couplings (21), whereas δ, α and β bands all show similar connection densities (25, 23 and 23 significant couplings, respectively).

## 4. Discussion and conclusion

Aiming at investigating if Brain–Heart Interplay (BHI) extends onto the multifractal (MF) domain, we devised a novel signal processing framework to perform a quantitative functional analysis of BHI in the MF domain using a non-Gaussian expansion. Functional coupling is estimated between MF features from brain and heartbeat dynamics using MIC, with MF spectra estimated using the wavelet p-leaders MF formalism. The proposed methodology was evaluated and tested for experimental EEG and HRV series gathered from 24 healthy subjects at a resting state and during a CPT.

The proposed processing pipeline leverages previous findings on brain and heartbeat dynamics coupling estimated using an MIC [7] and the intrinsic MF nature of heartbeat and EEG dynamics [15,21], demonstrating non-Gaussian and nonlinear behaviour [27,28]. It was also suggested that a complete MF analysis of heartbeat dynamics should incorporate inhomogeneous point-process models [27,38].

The results show that synchronized changes in brain and heartbeat dynamics occur in the MF domain for the CPT phase, demonstrating that changes in the functional BHI within the MF domain occur in response to certain sympathovagal changes. This is particularly evident for the second-order MF cumulant C2, and for the first two indices of the non-Gaussian expansion (i.e. LQ1 and LQ2), suggesting that major differences are associated with the nonlinear dynamics features of HRV and EEG parameter dynamics. This hypothesis is further supported by the fact that the first-order MF cumulant (C1), which stems from linear behaviour in the time domain, detected few significant BHI differences associated with HRV linear dynamics frequency quantifiers. Of note, first and third non-Gaussian MF indexes (i.e. LQ1 and LQ3) show differences in the RR- and HR-variance. This might be due to the nonlinear relation between RR and HR, whose effects on HRV power and variance have been previously characterized [49,50]. More specifically, LQ1 is associated with the quantification of general departure from Gaussianity and LQ2 is associated with the asymmetry of the distribution. As a consequence, we suggest that the non-Gaussian features of EEG- and HRV-derived series should be considered when investigating BHI dynamics.

In previous studies, we proved that a strong sympathovagal elicitation as the CPT generally leads to less pronounced MF behaviour separately in the EEG and HRV time series [27,28], as well as in the linear interplay between the two electrophysiological series, particularly in the heart-to-brain direction and the HRV-LF and EEG-γ frequency bands [12].

Here, more pronounced differences between the two experimental conditions are evident at higher EEG bands, particularly the γ range, and, for the cardiovascular system, the HRV-LF band and bispectral features. The results presented here confirm the complexity of the BHI phenomenon and, speculatively, we suggest that this arises from multiple responses associated with timing, scalp regions and directionality, which underlies bodily reactions to the allostatic state in order to re-establish homeostasis.

It should be noted that the proposed framework contains few limitations. First, the use of MIC does not allow the directionality of the functional brain–heart coupling to be assessed; the MIC, in fact, does not allow for such a causal inference, although it assesses statistical independence and quantifies linear and nonlinear couplings between the system outputs. Second, the statistical power of the comparison between experimental sessions is dependent on the number of EEG sensors, thus limiting the investigation of specific brain areas associated with cardiac interplay.

To the best of our knowledge, this study is the first to extend the quantitative assessment of functional BHI to the MF domain.

We conclude that the co-occurrence of MF behaviours between brain and heartbeat dynamics does exist, namely the functional BHI in the MF domain through cumulants and non-Gaussian expansions, and that they vary following sympathovagal changes induced by peripheral stress, such as that experienced during a CPT.

Future research efforts will be directed towards the application of the proposed framework to experimental datasets gathered both from healthy subjects and patients with brain and/or cardiovascular disorders. Consequently, we should be able to extract further valuable insights into the influences of different sympathovagal changes as well as cognitive/affective elicitation.

## Data accessibility

The informed consent forms signed by the subjects prevent data from being publicly available. Data may be requested via mail by researchers, upon reasonable request and verification of all ethical aspects, at: vincenzo.catrambone@ing.unipi.it.
